# Delayed diagnosis of persistent Q fever: a case series from China

**DOI:** 10.1186/s12879-024-09484-w

**Published:** 2024-06-17

**Authors:** Shanshan Wang, Ke Xu, Gang Wang

**Affiliations:** Department of Infectious Disease, Cheeloo College of Medicine, Qilu Hospital, Shandong University, Jinan, Shandong 250012 China

**Keywords:** Q fever, Coxiella burnetii, Metagenomic next-generation sequencing

## Abstract

**Background:**

Q fever, caused by the zoonotic pathogen Coxiella burnetii, exhibits a worldwide prevalence. In China, Q fever is not recognized as a notifiable disease, and the disease is overlooked and underestimated in clinical practice, leading to diagnostic challenges.

**Case presentation:**

We present a case series of three patients diagnosed with persistent Q fever between 2022 and 2023. The average age of our three cases was 63.33 years old, consisting of two males and one female. The medical history of the individuals included previous valve replacement, aneurysm followed by aortic stent-graft placement and prosthetic hip joint replacement. At the onset of the disease, only one case exhibited acute fever, while the remaining two cases were devoid of any acute symptoms. The etiology was initially overlooked until metagenomic next-generation sequencing test identified Coxiella burnetii from the blood or biopsy samples. Delayed diagnosis was noted, with a duration ranging from three months to one year between the onset of the disease and its confirmation. The epidemiological history uncovered that none of the three cases had direct exposure to domestic animals or consumption of unpasteurized dairy products. Case 1 and 2 resided in urban areas, while Case 3 was a rural resident engaged in farming. All patients received combination therapy of doxycycline and hydroxychloroquine, and no recurrence of the disease was observed during the follow-up period.

**Conclusion:**

Q fever is rarely diagnosed and reported in clinical practice in our country. We should be aware of persistent Q fever in high-risk population, even with unremarkable exposure history. Metagenomic next-generation sequencing holds great potential as a diagnostic tool for identifying rare and fastidious pathogens such as Coxiella burnetii.

**Supplementary Information:**

The online version contains supplementary material available at 10.1186/s12879-024-09484-w.

## Background

Q fever, caused by the fastidious bacterium Coxiella burnetii (C. burnetii), is a significant global zoonotic disease. It has been a longstanding public health concern with outbreaks being reported in numerous countries [1].

Persistent Q fever often develops following primary infection with C. burnetii, presenting as a localized infection [2]. The most prevalent complications are cardiovascular infections, such as endocarditis and vascular infections, resulting in elevated rates of mortality and morbidity [3, 4].

In China, Q fever continues to be unrecognized as a notifiable disease and receives few emphases [5]. Here, we report three cases of persistent Q fever with prolonged disease durations, presenting notable diagnostic complexities. The definitive diagnosis was achieved through detection of the causative pathogen C. burnetii using metagenomic next-generation sequencing (mNGS) testing. The delayed diagnosis of persistent Q fever can be attributed to multiple factors, with the most important one being a lack of awareness about Q fever.

## Case presentation

### Case 1

A 72-year-old female presented with progressive exertional chest distress, malaise and intermittent cough for three months in June 2022. She had a previous history of mitral valve replacement and tricuspid valve repair surgery in 2020.

Physical examination revealed normal vital signs. A grade 3 out of 6 holosystolic ejection murmur was heard in the auscultatory mitral area. The initial laboratory tests showed mild anemia with a hemoglobin level of 91 g/L (normal range, 130–175 g/L), a raised B-type natriuretic peptide value of 1018 pg/mL (normal range, ≤ 900 pg/mL) and normal hepatic function. Inflammatory markers revealed an elevated C-reactive protein (CRP) level of 25 mg/L (normal range, 0-10 mg/L) and an erythrocyte sedimentation rate (ESR) level of 51 mm/h (normal range, 0–15 mm/h). Computed tomography (CT) findings revealed bilateral pneumonia and moderate pleural effusion. With a 6 × 3 mm vegetation detected on the prosthetic valve by an echocardiography, the possible diagnosis of infective endocarditis (IE) was suggested. She was empirically treated with intravenous vancomycin (1000 mg every 12 h) after blood cultures taken. Meanwhile, serology for Brucella, serum galactomannan and 1,3-beta-D-glucan assays were all negative. With no confirmed etiology, an mNGS test was performed using the Illumina Nextseq 550 sequencing platform (Illumina, San Diego, USA). The detail methodology related to mNGS was presented in Supplementary Materials (Supplementary methodology of mNGS). Subsequently, the result indicated the existence of C. burnetii (mapping sequence number 590, relative abundance 63.85%) (Supplementary Fig. 1). Her epidemiological history revealed that she lived in an urban area with no contacting with any domestic animals. She also denied having consuming unpasteurized dairy products. Serology test of Q fever was performed and the result of immunofluorescence assay (IFA) (an in-house assay performed at the Bacterial disease laboratory of Jinan Center for Disease Control and Prevention) showed a phase I IgG titer of 1600, a phase II IgG titer of 9.24, consistent with persistent C. burnetii infection. With the final diagnosis of Q fever endocarditis, she was treated with oral doxycycline and hydroxychloroquine.

At the 2- month follow-up, the alleviation of all symptoms coincided with the improvement in inflammation markers and the resolution of pleural effusion. 18 months after discharge, no signs of vegetation were observed on echocardiography and the regimen has been continued until now.

## Case 2

A 61-year-old man was admitted with recurrent fever and abdominal pain in July 2023. He had a history of tobacco and alcohol use but quit in 2019 after undergoing a lobectomy for early-stage lung cancer.

He developed a high-grade fever with chills and malaise in January 2023. The fever was alleviated through intravenous fluoroquinolones intermittently. Two months later, the patient presented with progressive abdominal pain. An aortic computerized tomography angiography (CTA) revealed the presence of a pseudoaneurysm at the junction of the thoracoabdominal aorta, suggesting potential infectious lesions. In March 2023, he underwent endovascular aneurysm repair (EVAR) with the placement of one aortic stent. With no causative pathogen identified, oral amoxicillin was prescribed empirically after discharge. However, he continued to experience recurrent fever and abdominal pain over the following two months.

**Fig. 1:**
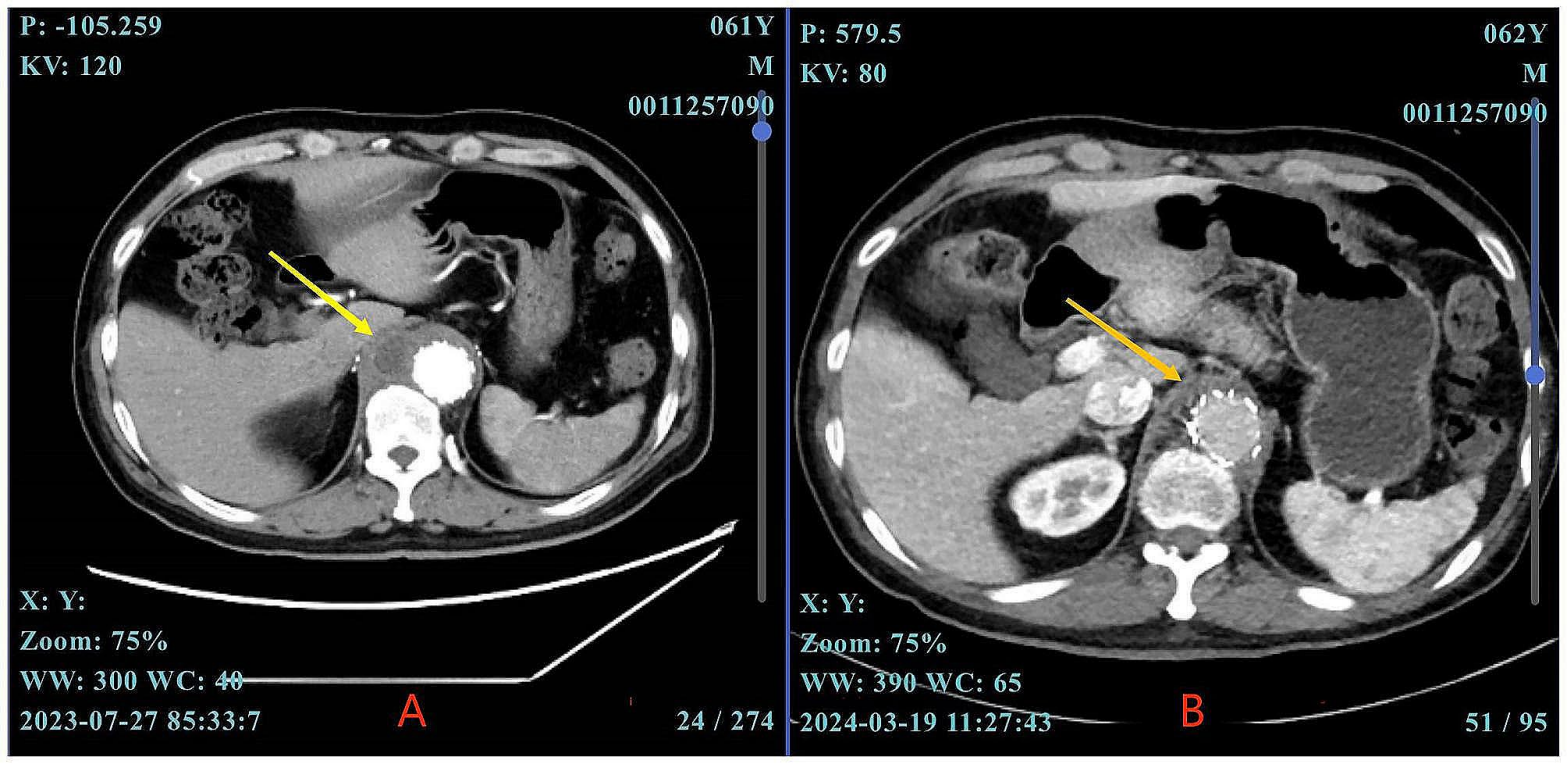
The abdominal CTA of case 2. **(A)** an annular low-density focus on the right side of the thoraco-abdominal aortic stent (yellow arrows). **(B)** a noticeable reduction in the size of the necrotic focus (orange arrows)

At admission, physical examinations revealed tenderness in the upper abdomen upon palpation. The laboratory findings showed a white blood cell count (WBC) of 10.75 × 10^9^/L (normal range, 3.50 × 10^9^ − 9.50 × 10^9^ /L), an ESR of 66 mm/h, and a CRP of 41.01 mg/L. As no pathogen identified in the blood, the empirical treatment was initiated, consisting of intravenous administration of vancomycin (1000 mg every 12 h) and meropenem (1 g every 8 h). However, the symptoms did not improve and repeated CTA revealed an annular low-density focus on the right side of the aortic stent (Fig. 1A). Subsequent 18 F-fluorodeoxyglucose positron emission tomography /CT confirmed the presence of the same mass exhibiting heterogeneously high fluorodeoxyglucose uptake, indicative of infectious lesions. A CT-guided biopsy of the mass yielded pus, which was subjected to bacterial culture, staining, and mNGS testing. The mNGS analysis revealed C. burnetii with a mapping sequence number of 269 and a relative abundance of 93.4% (Supplementary Fig. 2). The patient was a retired individual who resided in an urban environment. He had an unremarkable epidemiological history of livestock exposure. Enzyme-linked immunosorbent assay (ELISA) (an in-house assay performed at the Bacterial disease laboratory of Jinan Center for Disease Control and Prevention) showed positive result of antiphase I C. burnetii IgG antibody. With the diagnosis of persistent C. burnetii vascular infection, he was treated with oral doxycycline and hydroxychloroquine, and then discharged with a long-term medication plan. At the 8-month follow-up, complete remission of symptoms was achieved with a noticeable reduction in the size of the necrotic focus observed by a repeat CTA scan (Fig. 1B).

## Case 3

A 57-year-old farmer presented with intermittent left hip pain in December 2023. He had a left artificial femoral head replacement in 2018. Recently, he was diagnosed with osteomyelitis of the mandibular condyle and underwent surgical debridement of the necrotic bone. With negative culture result, he was empirically treated with intravenous moxifloxacin.

**Fig. 2:**
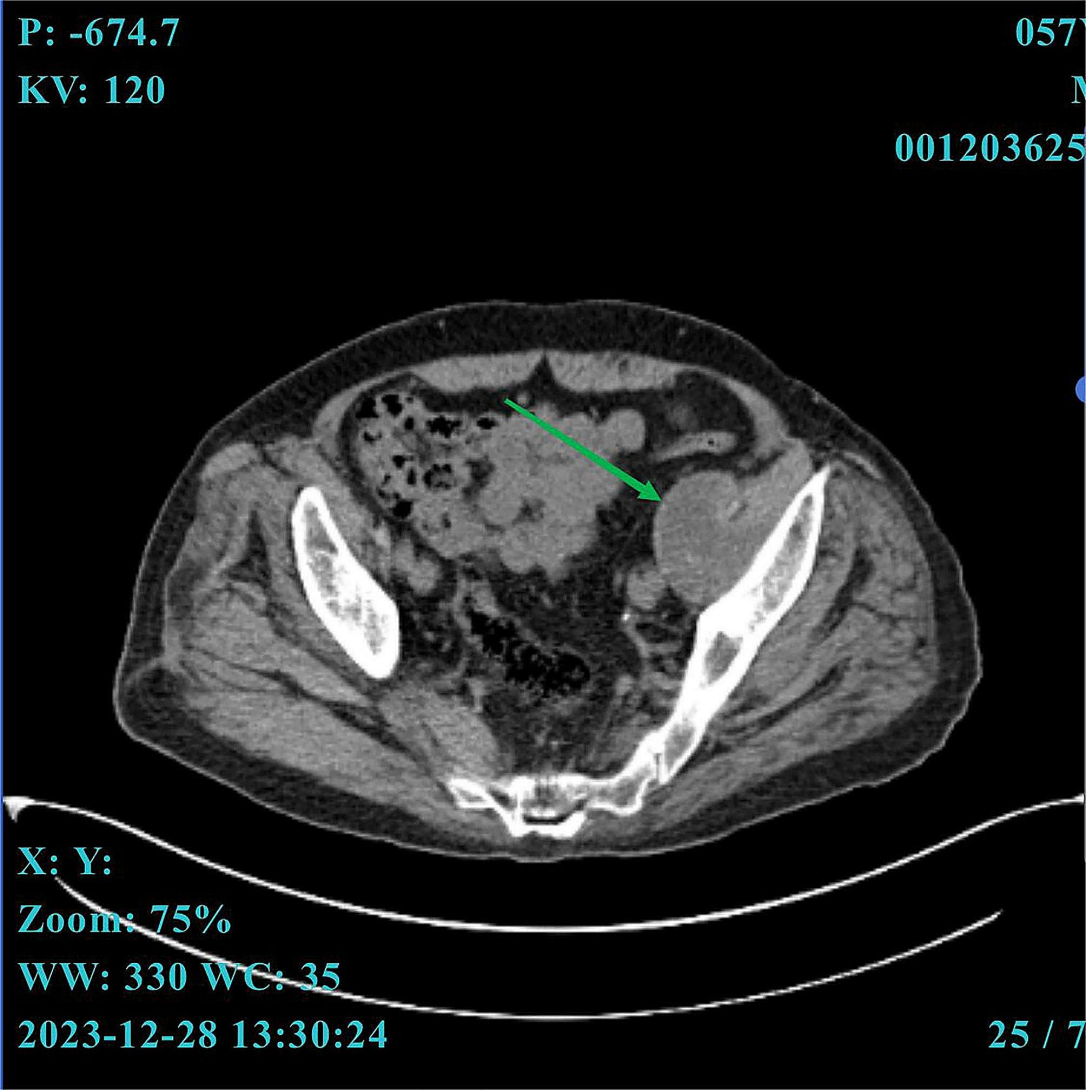
The pelvic CT scan of case 3. An anomalous density mass situated in the left iliopsoas region (green arrows)

Upon admission, his physical examination demonstrated normal vital signs. Laboratory tests revealed mild anemia with a hemoglobin of 106 g/L and a normal WBC count. Inflammation markers indicated normal levels of CRP and PCT, but an elevated ESR value of 57 mm/h. The gamma-glutamyl transferase exhibited a slight increase to 161 U/L (normal range, 10–60 U/L) and alkaline phosphatase increased to 166 U/L (normal range, 45–125 U/L). No organism was isolated from the repeated blood cultures. A pelvic CT scan revealed an anomalous density mass in the left iliopsoas region (Fig. 2). Next, the mass was biopsied with CT guidance. The aspirated fluid sample underwent routine microbiological testing and mNGS analysis. 24 h later, the mNGS reported that C. burnetti was detected with the mapping sequence number of 311 and the relative abundance of 98.73% (Supplementary Fig. 3). While the routine culture and staining yielded negative results.

The ELISA testing of Q fever (an in-house assay performed at the Bacterial disease laboratory of Jinan Center for Disease Control and Prevention) demonstrated positive result for anti-C. burnetii phase I IgG antibody. The epidemiological history revealed he had no clear record of direct exposure to livestock or their products. He was diagnosed with C. burnetii prosthetic joint infection (PJI) and initiated a prolonged regimen of oral doxycycline and hydroxychloroquine. During the most recent mobile phone follow-up in March 2024, the patient reported a reduction in left hip pain and complete healing of the wound on the right jaw.

## Discussion and conclusions

Q fever, caused by bacterium C. burnetii, is a global zoonosis of great importance and public health concern [1]. In China, Q fever has been significantly overlooked and underestimated as a non-notifiable disease, despite its wide distribution indicated by previous seroepidemiological studies [6].

C. burnetii has the potential to infect a wide range of animal species, with domestic ruminants serving as the major reservoirs for human transmission [7]. Infection commonly occurs through the inhalation of aerosols contaminated by asymptomatic animals’ products and excreta. This unique transmission mode complicates the identification of populations at risk without direct exposure. None of our three cases had clear epidemiological connections to Q fever, as none reported direct contact with livestock. It is worth noting that the patient in Case 3 is a farmer, an occupation recognized as high-risk for Q fever but often neglected, thus requiring additional investigation [8].

The clinical manifestations of Q fever are nonspecific and diverse. Primary infection may be asymptomatic, or present with acute onset of fever, accompanied by pneumonia, hepatitis or other rare manifestations [7]. Due to its similarity to acute respiratory infectious disease, Q fever is frequently underdiagnosed and should be considered as a significant differential diagnosis for febrile illness [9].

Following primary infection, C. burnetii can establish persistent focalized infections by evading the immune system and replicating covertly within specific anatomical sites [10]. Less than 5% of cases with primary infection may progress into persistent infection [11]. Advanced age was identified as one of the major risk factors for the development of persistent Q fever [12]. The average age of our three cases exceeded 60 years old. Endocarditis and vascular infections are the frequently involved forms, carrying a high mortality rate [7, 13]. Underlying valvular heart disease emerges as the most significant risk factor for the progression to Q fever endocarditis, which often presents with subtle vegetations in its initial stages that may evade detection on echocardiography [14]. Q fever vascular infections are found to be associated with a worse prognosis when compared to endocarditis. Both aneurysms and aortic graft implantation independently increase the risk of vascular infection [15]. In case 2, the exact role of C. burnetii in the development of aneurysms or infection following arterial stent implantation remained uncertain due to the absence of initial pathogen testing for C. burnetii. The occurrence of PJI is also a concern as a devastating complication for joint replacement [16]. C. burnetii is often overlooked as a causative pathogen for PJI, as Staphylococcus species are considered the predominant pathogens [17]. The symptoms for C. burnetii PJI may remain mild and insidious for an extended period without specific manifestations [16].

Due to the highly infectious and fastidious nature of C. burnetii, the diagnosis of Q fever has long been relied on serology [18]. Common serology methods include IFA and ELISA, with IFA considered the reference method and ELISA as a convenient alternative but with lower sensitivity [2, 18]. High levels of Q fever phase I antibodies often indicate of a persistent infection [2]. A phase I IgG antibody titer > 1:800 is recommended as a major microbiological criterion for diagnosing Q fever endocarditis [19, 20]. However, the serology is not always reliable due to certain strains of C. burnetii displaying a high level of phase I IgG titers during the acute phase, while low serological titers may be observed in cases of persistent infection [10]. Therefore, the establishment of an appropriate cut-off value for phase I IgG antibodies to distinguish between past and persistent infection remains a challenge [21]. Another common diagnostic tool for C. burnetii is the polymerase chain reaction test (PCR), which allows for direct identification of the bacterium in both acute and persistent infection phases [7]. However, its accuracy may be affected by previous antibiotic use [14]. Both serology tests and PCR, as targeted diagnostic methods, are primarily conducted in cases with a strong clinical suspicion of Q fever.

It is extremely difficult to make a definitive diagnosis of persistent Q fever. In our three cases, the duration from the initial onset of the disease to the final diagnosis ranged from three months to one year. The delayed diagnosis may primarily be attributed to a lack of awareness of Q fever, as well as atypical manifestations, limited epidemiological information, and unavailability of routine diagnostic tools in most healthcare facilities in our nation.

Timely identification of the etiology of persistent Q fever is essential for effective treatment [8, 13]. mNGS, characterized by its unbiased and high-throughput sequencing capabilities, is employed in cases where the infectious etiology is unclear and may associate with a diverse range of possible microorganisms [22]. It can rapidly detect almost all the organisms present in clinical samples, thereby overcoming the limitation of only being able to test specific targets using PCR [23].With the decreasing cost, there has been an increasing utilization of mNGS in diagnosing complex infectious diseases and febrile illnesses caused by unknown pathogens. In 2004, a neuroleptospirosis was diagnosed in an immunosuppressed patient with prolonged febrile illness using mNGS [24]. Since then, more studies have been conducted to assess its microbial identification capabilities. It was suggested mNGS had a higher sensitivity for pathogen identification, and unlike PCR, it is less impacted by prior empirical antibiotic exposure [25]. The inclusion of mNGS for the detection of C. burnetii has been added as one major microbiological criterion in diagnosing Q fever endocarditis in 2023 [20].

Two significant challenges encountered during the sequencing process are effectively eliminating human sequences and preventing potential microbial contamination. Additionally, there is a lack of standardized protocols or guidelines for the interpretation of mNGS findings. For intracellular bacteria such as C. burnetii, a positive result is reported when at least one read is successfully mapped to the species or genus level, considering the difficulty associated with DNA extraction [26]. The widespread adoption of mNGS in clinical settings has been hindered by its high cost and the need for specialized personnel [27]. Consequently, mNGS is utilized as a last resort rather than a replacement for routine microbiological testing.

In conclusion, we report a case series of persistent Q fever diagnosed through mNGS and verified by serology assays. The diagnosis of Q fever was initially overlooked until the presence of C. burnetii was revealed through mNGS testing. Through our study, mNGS can be a helpful tool for rapid and precise diagnosis of complex infections, such as persistent Q fever [26]. Meanwhile, it is essential to conduct large epidemiological surveys to accurately determine the real prevalent rate of Q fever in our country.

### Electronic supplementary material

Below is the link to the electronic supplementary material.

 Supplementary methodology of mNGS

 Supplementary Fig. 1

 Supplementary Fig. 2

 Supplementary Fig. 3

## Data Availability

All data generated or analysed during this study are included in this published article.

## Abbreviations

C. burnetii: Coxiella burnetii
CRP: C-reactive protein
ESR: Erythrocyte sedimentation rate
IE: infective endocarditis
CT: computed tomography
mNGS: metagenomic next-generation sequencing
PCR: polymerase chain reaction test
IFA: immunofluorescence assay
WBC: white blood cell count
CTA: computerized tomography angiography
ELISA: Enzyme-linked immunosorbent assay
PJI: prosthetic joint infection
