# Supplementary material for: Delayed diagnosis of persistent Q fever: a case series from China
Source: BMC Infect Dis. 2024 Jun 17;24:591. doi: 10.1186/s12879-024-09484-w (PMC11181675; doi:10.1186/s12879-024-09484-w)
Supplement: Supplementary file 1 — Supplementary methodology of mNGS [file 12879_2024_9484_MOESM1_ESM.pdf]

Metagenomic Next-generation Sequencing and Analysis

Methods:

Nucleic Acid Extraction

The samples were sealed aseptically and stored at -20 ℃ to perform mNGS detection immediately. The DNA was extracted and purified by taking 200 uL of cell-free supernatant sample according to the instructions of QIAamp DNA Micro Kit (QIAGEN, Hilden, Germany). DNA concentration and quality were checked through Qubit 3.0 Fluoremeter (Invitrogen, Q33216) and agarose gel electrophoresis (Major Science, UVC1-1100).

Library Generation and Sequencing

DNA library construction was performed according to the Qiagen library construction kit (QIAseq Ultralow Input Library Kit) operating instructions. Library quality control was performed by Qubit 3.0 Fluoremeter (Invitrogen, Q33216) and Agilent 2100 Bioanalyzer (Agilent Technologies, Palo Alto, USA). Qualified DNA libraries with different barcode tags were pooled and then sequenced using the Illumina Nextseq 550 sequencing platform (Illumina, San Diego, USA) and a SE75bp sequencing strategy.

Bioinformation Pipeline

After obtaining the sequencing data, high quality data was generated by filtering out connectors, low quality, low complexity and shorter sequences. Next human-derived sequences matching to the human reference database (hg38) were removed by using SNAP software. The remaining data were then aligned to the microbial genome database using Burrow-Wheeler Alignment. This database contains a large collection of microbial genomes from NCBI containing more than 30,000 microorganisms, including 17,748 species of bacteria, 11,058 species of viruses, 1,134 species of fungi, and 308 species of parasites. The microbial composition of the samples was finally determined. The positive criteria for the mNGS result were set as follows:

1) For bacteria other than TB, fungi other than Cryptococcus and parasites: sequencing coverage in the top 10 of all pathogens detected and not detected in the negative control (NTC); or sample/NTC with an RPM (reads per million mapped reads) ratio greater than 10.

(2) For viruses, tuberculosis, cryptococci, rickettsia app. and other intracellular bacteria: at least 1 specific sequence was detected and not detected in the NTC; or the RPM ratio of sample/NTC was greater than 5.
