# Supplementary figures and images for: Delayed diagnosis of persistent Q fever: a case series from China
Source: BMC Infect Dis. 2024 Jun 17;24:591. doi: 10.1186/s12879-024-09484-w (PMC11181675; doi:10.1186/s12879-024-09484-w)

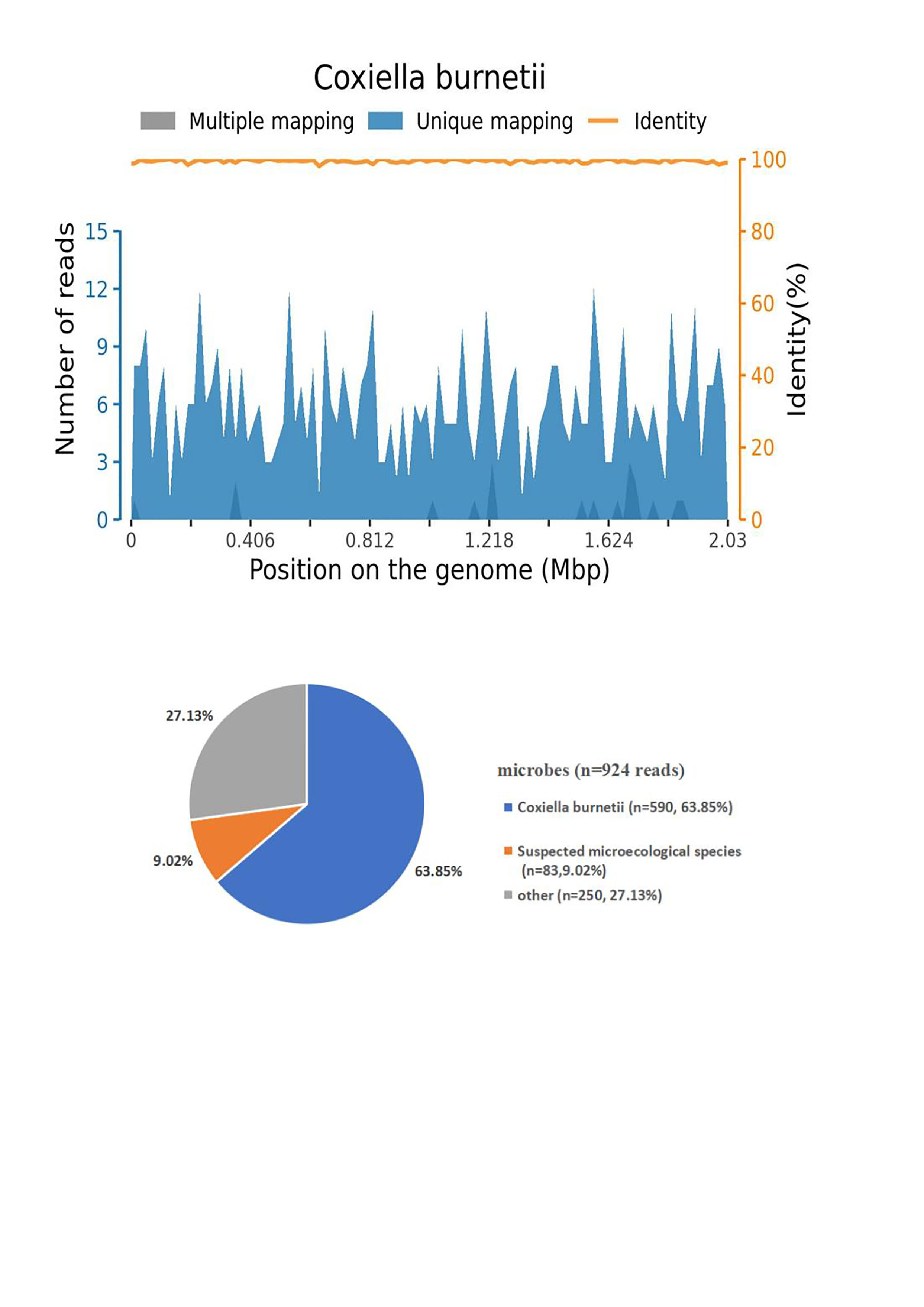

Supplement: Supplementary file 2 — Supplementary Fig. 1 [file 12879_2024_9484_MOESM2_ESM.jpg]

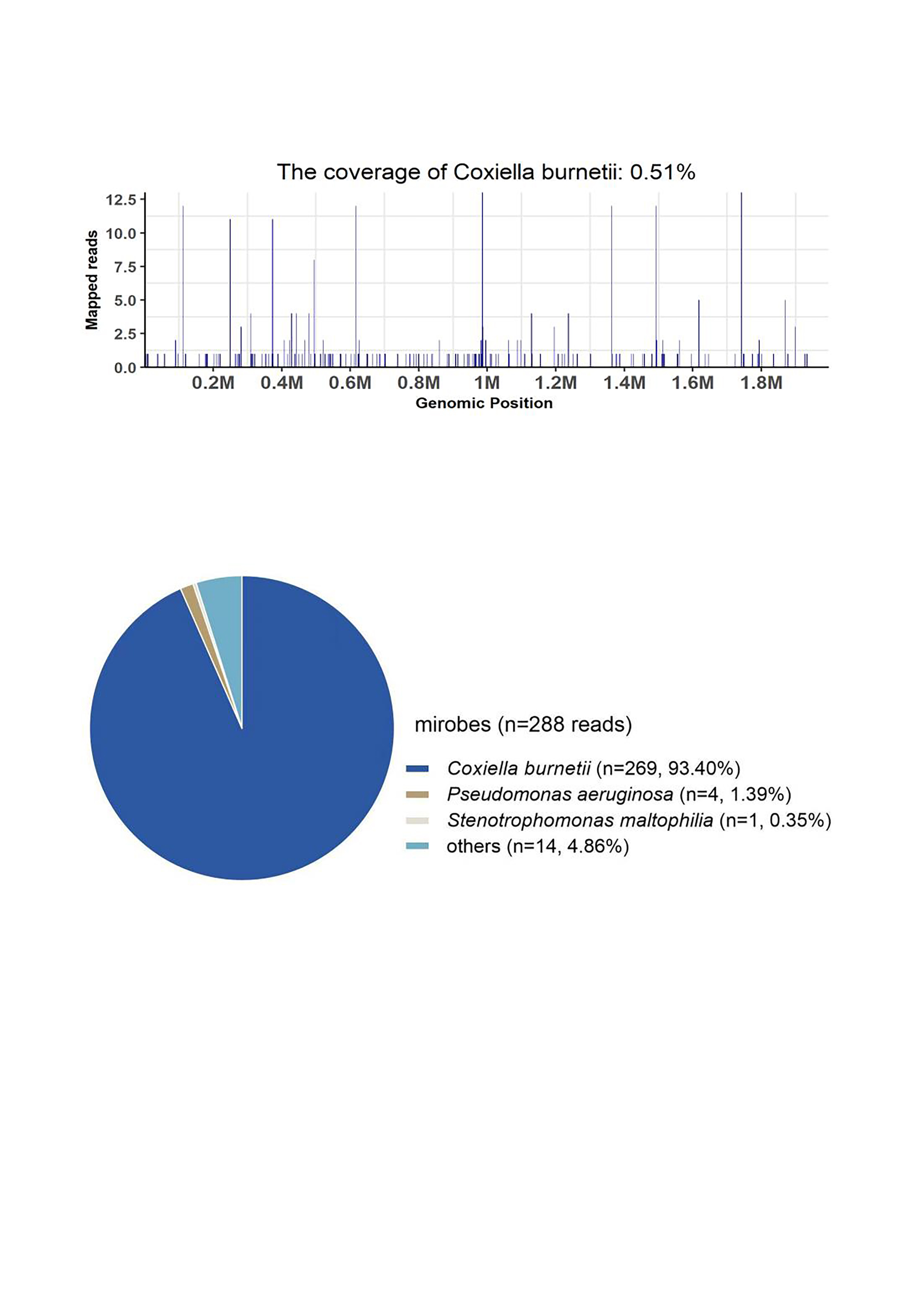

Supplement: Supplementary file 3 — Supplementary Fig. 2 [file 12879_2024_9484_MOESM3_ESM.jpg]

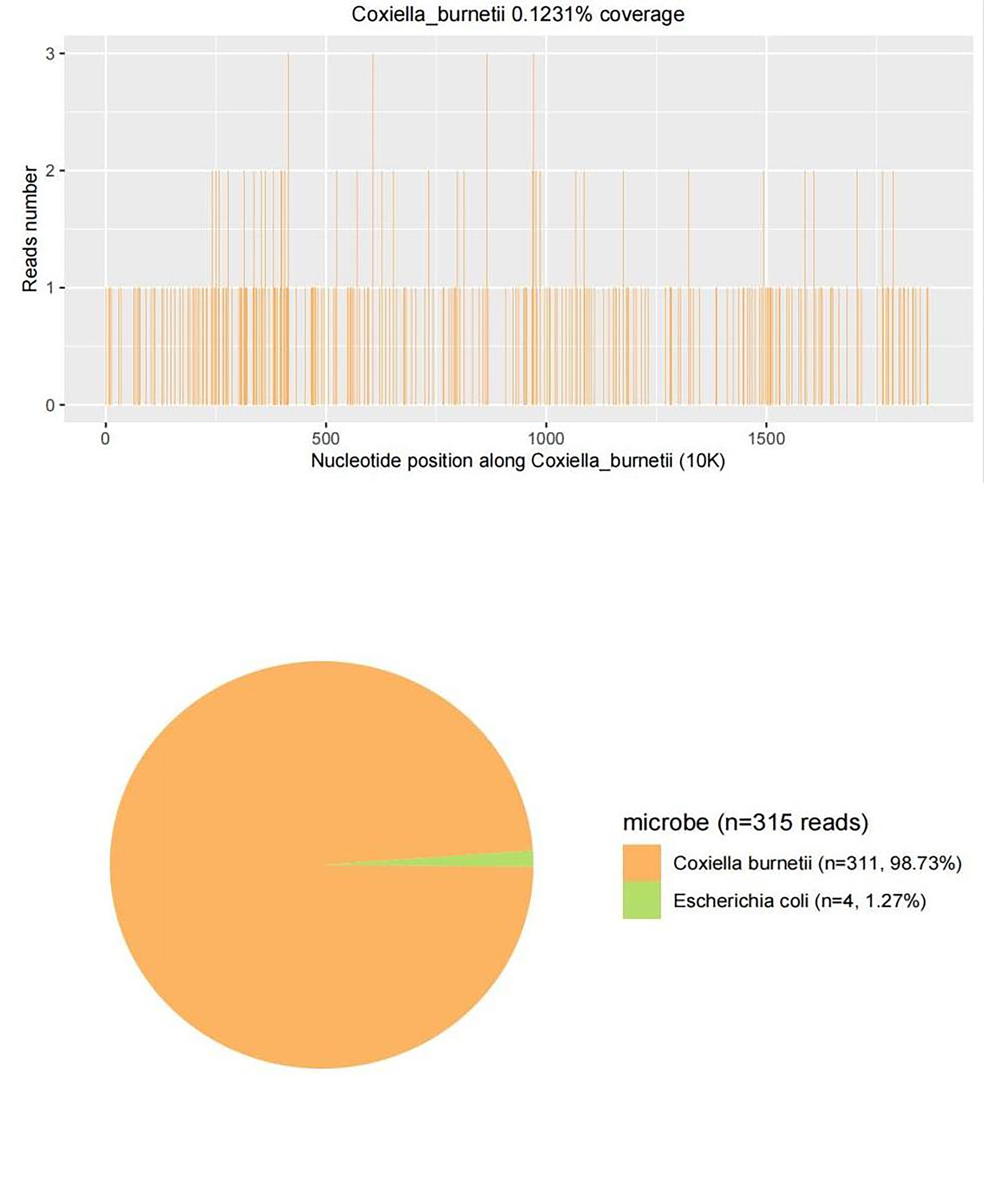

Supplement: Supplementary file 4 — Supplementary Fig. 3 [file 12879_2024_9484_MOESM4_ESM.jpg]
